# Low Serum 25-Hydroxyvitamin D Levels Are Related to Frailty and Sarcopenia in Patients with Chronic Liver Disease

**DOI:** 10.3390/nu12123810

**Published:** 2020-12-12

**Authors:** Chisato Saeki, Tomoya Kanai, Masanori Nakano, Tsunekazu Oikawa, Yuichi Torisu, Masayuki Saruta, Akihito Tsubota

**Affiliations:** 1 Division of Gastroenterology and Hepatology, Department of Internal Medicine, The Jikei University School of Medicine, 3-25-8 Nishi-shimbashi, Minato-ku, Tokyo 105-8461, Japan; tomoyaaust@hotmail.com (T.K.); masanori-nakano@jikei.ac.jp (M.N.); oitsune@jikei.ac.jp (T.O.); torisu@jikei.ac.jp (Y.T.); m.saruta@jikei.ac.jp (M.S.); 2 Division of Gastroenterology, Department of Internal Medicine, Fuji City General Hospital, 50 Takashima-cho, Fuji-shi 417-8567, Shizuoka, Japan; 3 Core Research Facilities, Research Center for Medical Science, The Jikei University School of Medicine, 3-25-8 Nishi-shimbashi, Minato-ku, Tokyo 105-8461, Japan

**Keywords:** chronic liver disease, sarcopenia, frailty, vitamin D

## Abstract

Low vitamin D status is related to frailty and/or sarcopenia in elderly individuals. However, these relationships are unclear in patients with chronic liver disease (CLD). This study aimed at exploring the relationship between serum 25-hydroxyvitamin D [25(OH)D] levels and frailty or sarcopenia in 231 patients with CLD. Frailty was determined based on five factors (weight loss, low physical activity, weakness, slowness, and exhaustion). Sarcopenia was diagnosed by applying the Japan Society of Hepatology criteria. The patients were classified into three groups according to baseline 25(OH)D levels: low (L), intermediate (I), and high (H) vitamin D (VD) groups. Of the 231 patients, 70 (30.3%) and 66 (28.6%) had frailty and sarcopenia, respectively. The prevalence rate of frailty and sarcopenia significantly increased stepwise with a decline in the vitamin D status. The L-VD group showed the highest prevalence rates of frailty and sarcopenia (49.1% (28/57), *p* < 0.001 for both), whereas the H-VD group showed the lowest prevalence rates of frailty (15.3% (9/59)) and sarcopenia (18.6% (11/59)) (*p* < 0.001 for both). Multivariate analysis identified serum 25(OH)D levels as a significant independent factor related to frailty and sarcopenia. Serum 25(OH)D levels significantly correlated with handgrip strength, skeletal muscle mass index, and gait speed. In conclusion, low serum vitamin D level, especially severe vitamin D deficient status, is closely related to frailty and sarcopenia in patients with CLD.

## 1. Introduction

Sarcopenia, characterized by a generalized decline of skeletal muscle mass and strength, is a serious complication in patients with chronic liver disease (CLD) [1,2]. Frailty is defined as increased vulnerability to stressors due to multisystem physiological impairment and is associated with, but not equivalent to, sarcopenia [3,4,5]. Sarcopenia and frailty negatively impact the quality of life, resulting in disability, and increase the risk of institutionalization and mortality [5,6,7,8,9]. In general, poor nutritional status, including low levels of branched-chain amino acids (BCAAs), vitamin D, zinc, and long-chain omega-3 fatty acids, is considered one of the causes of sarcopenia and frailty [10,11]. Therefore, nutritional evaluations and early therapeutic interventions for these disorders are imperative in patients with CLD.

The liver plays a crucial role in the metabolism of vitamin D, which is included in a group of fat-soluble secosteroids. Vitamin D_3_ (cholecalciferol) and D_2_ (ergocalciferol) are synthesized in the skin under ultraviolet exposure and ingested from the diet and/or supplements. These two vitamin D forms are inactive until they are hydroxylated in the liver to form 25-hydroxyvitamin D [25(OH)D]. 25(OH)D is the main circulating form of vitamin D and is used as a representative biomarker of a person’s vitamin D status. Subsequently, 25(OH)D undergoes further hydroxylation in the kidneys to form active 1,25-dihydroxyvitamin D [1,25(OH)D] [12]. Therefore, a reduction in circulating vitamin D levels reflect an impaired liver function or malnutrition. In addition, bile production is impeded in patients with CLD, leading to decreased fat absorption, and consequently, impaired uptake of vitamin D [13]. Accordingly, patients with CLD are frequently accompanied by low vitamin D status; vitamin D deficiency (≤20 ng/mL) occurs more frequently among patients with CLD (47–78%) than in the general population (34–59%) [12,14,15,16,17,18,19,20,21]. Importantly, vitamin D deficiency causes high mortality in patients with liver cirrhosis (LC), irrespective of the impact of hepatocellular carcinoma [16,22].

Recently, several investigators have focused on the association between 25(OH)D levels and skeletal muscle function [23,24]. Previous studies have demonstrated that low vitamin D levels were related to sarcopenia, frailty, impaired physical performance, and an increased risk of mortality in elderly individuals [25,26,27,28,29,30,31,32]. In addition, a recent study affirmed that low vitamin D levels were associated with sarcopenia in patients with CLD [15]. However, the relationship between 25(OH)D levels, sarcopenia, and frailty in patients with CLD has not yet been reported and thus, remains unknown. Therefore, in the present study, we aimed at exploring the relationship between serum 25(OH)D levels and the prevalence of sarcopenia and frailty in patients with CLD.

## 2. Patients and Methods

### 2.1. Study Design and Patients

The present cross-sectional study included 231 consecutive patients in whom CLD was diagnosed at Fuji City General Hospital (Shizuoka, Japan) between 2017 and 2020. The inclusion criteria were as follows: (1) the presence of CLD; (2) the availability of data on skeletal muscle mass index (SMI) measured using bioimpedance analysis (InBody S10; InBody, Seoul, Korea), and grip strength using a dynamometer (T.K.K5401 GRIP-D; Takei Scientific Instruments, Niigata, Japan); and (4) the availability of frailty data evaluated based on the diagnostic criteria proposed by Fried et al. [33]. The exclusion criteria were as follows: (1) patients with refractory ascites or implants or undergoing hemodialysis, as previously described [2]; (2) patients with alcoholic liver disease because heavy alcohol consumption frequently leads to malnutrition, including vitamin D deficiency; and (3) patients who had been receiving vitamin D supplementation within 12 months before the date of entry. This study was conducted according to the criteria set by the 2013 revision of the Declaration of Helsinki. The Ethics Committee of Fuji City General Hospital approved this study (approval no. 156).

### 2.2. Diagnosis of Sarcopenia and Frailty

A diagnosis of sarcopenia was made based on the criteria published by the Japan Society of Hepatology [1]. Briefly, sarcopenia was defined as a physical condition with reduced handgrip strength (<18 kg for women and <26 kg for men) and muscle mass (SMI <5.7 kg/m^2^ for women and <7.0 kg/m^2^ for men). The SMI was computed from the total muscle mass of the four limbs divided by the square of height (kg/m^2^). Gait speed was measured using the 6-m walk test (low gait speed was defined as <1.0 m/s). A diagnosis of frailty was made using a validated screening tool based on the following five components [33,34]: (1) weight loss (≥2 kg during the last six months); (2) weakness (handgrip strength <18 kg for women and <26 kg for men); (3) exhaustion, which was determined based on an agreement to the question: “In the last two weeks, have you felt tired without a reason?”; (4) slowness (gait speed of <1.0 m/s); and (5) low physical activity, which was established based on negative answers to the following two questions: “Do you engage in moderate levels of physical exercise or sports aimed at health?” or “Do you engage in low levels of physical exercise aimed at health?”. Frailty was determined by three or more agreements [34].

### 2.3. Clinical and Laboratory Assessments

Serum was collected from each patient in an early morning fasting state. The following blood tests were measured using routine, conventional methods: prothrombin time-international normalized ratio (PT-INR), albumin, total bilirubin, and BCAA. The level of serum 25(OH)D, a biomarker of a person’s vitamin D status, was measured using a chemiluminescent immunoassay (Hitachi Chemical Diagnostics Systems, Tokyo, Japan). Vitamin D deficiency, insufficiency, and sufficiency were defined as serum 25(OH)D levels of ≤20.0 ng/mL, 20.1–29.9 ng/mL, and ≥30.0 ng/mL, respectively, based on the conventional classification of vitamin D status [35].

### 2.4. Provisional Reclassification Based on the Serum 25-Hydroxyvitamin D Levels

The median serum 25(OH)D level for all patients was 14.0 (interquartile range, 10.5–18.1) ng/mL. The patients were reclassified into three groups according to the first and third quartiles (Figure S1): (1) low 25(OH)D (L-VD) group with levels ≤10.5 ng/mL (first quartile); (2) intermediate 25(OH)D (I-VD) group with levels between 10.5 and 18.1 ng/mL (third quartile); and (3) high 25(OH)D (H-VD) group with levels ≥18.1 ng/mL.

### 2.5. Statistics

Categorical and continuous data are expressed as number (percentage) and median (interquartile range), respectively. The chi-squared test was performed to compare frequencies in categorical data between two groups. The Mann–Whitney U test was performed to compare differences in continuous data between two groups. The Kruskal–Wallis test followed by the Steel–Dwass post hoc test was performed for multiple continuous data comparisons among three groups. Univariate and multiple logistic regression analyses were done to determine significant and independent factors associated with sarcopenia and frailty. The Cochran–Armitage trend test was performed to evaluate whether a trend was present between one variable with two categories and one variable with multiple categories. The Spearman’s rank correlation test was used to analyze correlations between two continuous variables. These analyses were done using the SPSS software (ver. 26, IBM, Japan, Tokyo, Japan). A value of *p* < 0.05 was considered statistically significant.

## 3. Results

### 3.1. Baseline Characteristics of Patients

The baseline characteristics of the 231 CLD patients in the present study are shown in Table 1. The patients consisted of 95 men (41.1%) and 136 women (58.9%), with a median age of 70.0 (60.0–76.0) years for all patients. Ninety-eight (42.4%) patients were diagnosed with LC. The median 25(OH)D level was 14.0 (10.5–18.1) ng/mL. The median values of handgrip strength, SMI, and gait speed were 22.1 (17.4–29.4) kg, 6.30 (5.54–7.12) kg/m^2^, and 1.10 (0.91–1.25) m/s, respectively. Eighty-one (35.1%) patients had slow gait speed.

### 3.2. Comparison of Clinical Characteristics among Patients with and without Sarcopenia

Sixty-six (28.6%) were diagnosed with sarcopenia among the 231 patients (Table 1). The patients in the sarcopenia group were significantly older and had a lower body mass index (BMI) than those in the non-sarcopenia group (*p* < 0.001 for both). The sarcopenia group had a higher prevalence of LC than the non-sarcopenia group (60.6% vs. 35.2%; *p* < 0.001). As for the biochemical findings, the levels of albumin (*p* = 0.003), BCAA (*p* < 0.001), and 25(OH)D (*p* = 0.001) in the sarcopenia group were significantly lower than those in the non-sarcopenia group. Vitamin D deficiency was significantly more frequent in the sarcopenia group than in the non-sarcopenia group (*p* = 0.048). The sarcopenia group showed significantly higher rates of frailty (77.3% vs. 11.5%; *p* < 0.001) and slow gait speed (74.2% vs. 19.4%; *p* < 0.001) than the non-sarcopenia group.

### 3.3. Significant Factors Related to Sarcopenia among Patients with Chronic Liver Disease

The univariate analysis showed that the following eight variables were significantly associated with sarcopenia: age, BMI, LC, serum levels of albumin, BCAA, and 25(OH)D, PT-INR deficiency vitamin D (Table S1). Finally, the following four variables remained significant and independent on the multivariate analysis (Table 2): advanced age (odds ratio (OR), 1.087; 95% confidence interval (CI), 1.044–1.130; *p* < 0.001); the presence of LC (OR, 2.493; 95% CI, 1.180–5.266; *p* = 0.017); lower BMI (OR, 0.720; 95% CI, 0.635–0.817; *p* < 0.001); and lower 25 (OH)D levels (OR, 0.863; 95% CI, 0.794–0.937; *p* < 0.001).

### 3.4. Comparison of Clinical Characteristics among Patients with and without Frailty

The prevalence rate of frailty was 30.3% (70/231) (Table S2). The patients with frailty were significantly older and had a higher prevalence of LC (68.6% vs. 31.1%) and lower BMI than those without frailty (*p* < 0.001 for all). The patients in the frail group had significantly lower levels of 25(OH)D (*p* < 0.001), albumin (*p* < 0.001), BCAA (*p* < 0.001), and longer PT-INR (*p* = 0.014) than those in the non-frail group. The frail group had significantly higher rates of sarcopenia (72.9% vs. 9.3%; *p* < 0.001) and slow gait speed (92.9% vs. 9.9%; *p* < 0.001) than the non-frail group.

### 3.5. Significant Factors Related to Frailty among Patients with Chronic Liver Disease

On the univariate analysis, the following eight variables were significantly related to frailty: age, BMI, LC, serum levels of albumin, BCAA, and 25(OH)D, PT-INR, and vitamin D deficiency (Table S3). The following four variables remained significant and independent on the multivariate analysis (Table 3): advanced age (OR, 1.094; 95% CI, 1.052–1.138; *p* < 0.001); the presence of LC (OR, 3.749; 95% CI, 1.843–7.626; *p* < 0.001); lower BCAA levels (OR, 0.994; 95% CI, 0.990–0.998; *p* = 0.008); and lower 25(OH)D levels (OR, 0.887; 95% CI, 0.822–0.957; *p* = 0.002).

### 3.6. Prevalence of Sarcopenia and Frailty Defined according to the Conventional Classification of Vitamin D Status

**Figure 1:**
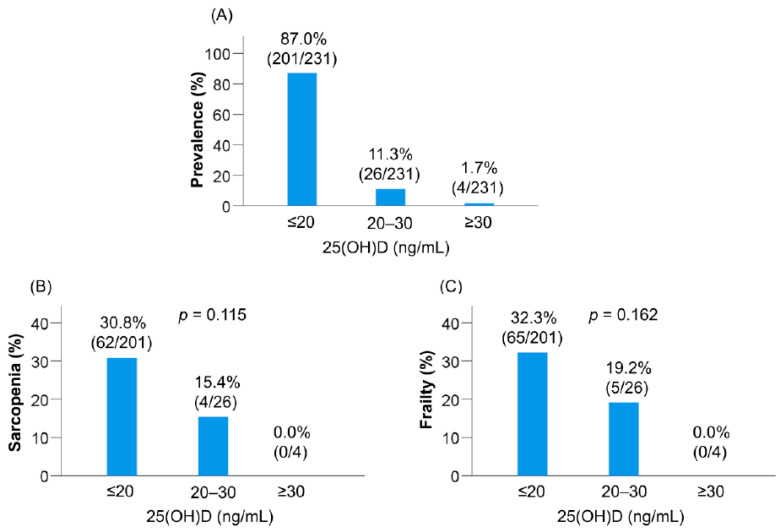
(**A**) Distribution of the serum 25-hydroxyvitamin D [25(OH)D] levels in patients with chronic liver disease. (**B**,**C**) The prevalence rates of sarcopenia and frailty based on the serum 25(OH)D levels.

Vitamin D deficiency, insufficiency, and sufficiency were present in 87.0% (201/231), 11.3% (26/231), and 1.7% (4/231) of the patients, respectively (Table 1, Figure 1A). The vitamin D deficient group had the highest prevalence of sarcopenia (30.8% (62/201), Figure 1B) and frailty (32.3% (65/201), Figure 1C) among the three groups, although the difference was not statistically significant.

### 3.7. Clinical Characteristics Based on the Provisional Reclassification of Vitamin D Status

**Figure 2:**
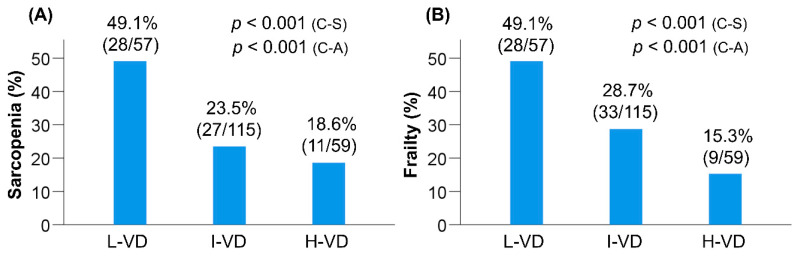
Comparison of clinical characteristics among the low vitamin D (L-VD), intermediate vitamin D (I-VD), and high vitamin D (H-VD) groups. (**A**) The L-VD group had the highest prevalence rate of sarcopenia (chi-squared test: *p* < 0.001), while the H-VD group had the lowest prevalence rate of sarcopenia (chi-squared test: *p* < 0.001) among the three groups. The prevalence of sarcopenia significantly increased in a stepwise manner with a decline in the serum 25-hydroxyvitamin D [25(OH)D] level (Cochran–Armitage trend test: *p* < 0.001). (**B**) The L-VD group had the highest prevalence rate of frailty (chi-squared test: *p* < 0.001), while the H-VD group had the lowest prevalence rate of frailty (chi-squared test: *p* < 0.001) among the three groups. The prevalence of frailty significantly increased in a stepwise manner with a decline in the 25(OH)D level (Cochran–Armitage trend test: *p* < 0.001). C-A, Cochran–Armitage trend test; C-S, chi-squared test.

As described previously, most patients (87.0%) had vitamin D deficiency (as defined by the conventional classification). Therefore, we could not confirm the statistical correlation between vitamin D deficiency/insufficiency and sarcopenia or frailty. To resolve this issue and explore the association between vitamin D status and disease conditions in more detail, we reclassified the patients into three groups provisionally according to the baseline serum 25(OH)D levels (as described in the Methods section). The distribution of the L-VD, I-VD, and H-VD groups was 24.7% (57/231), 49.8% (115/231), and 25.5% (59/231), respectively (Table 4). Gender (*p* = 0.012), BCAA (*p* < 0.001), SMI (*p* = 0.002), and handgrip strength (*p* < 0.001) were significantly different among these groups. Notably, the L-VD group had the highest prevalence rate of sarcopenia (49.1% (28/57); *p* <0.001; adjusted residual = |4.0|) and frailty (49.1% (28/57); *p* < 0.001; adjusted residual = |3.6|) among these groups, while the H-VD group had the lowest prevalence rate of sarcopenia (18.6% (11/59); *p* < 0.001; adjusted residual = |2.0|) and frailty (15.3% (9/59); *p* < 0.001; adjusted residual = |2.9|) (Table 4, Figure 2A,B). The prevalence rates of sarcopenia and frailty significantly increased in a stepwise manner with a decline in the 25(OH)D levels (*p* < 0.001 for both).

### 3.8. Correlation between Serum 25(OH)D Levels and Baseline Clinical Characteristics

**Figure 3:**
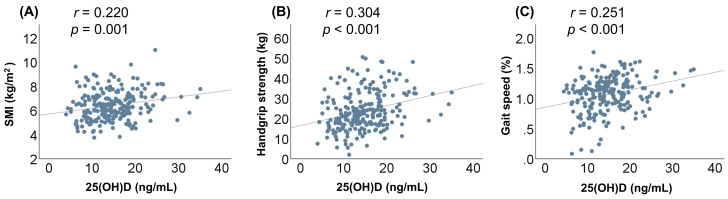
Correlations between the serum 25-hydroxyvitamin D [25(OH)D] levels and skeletal muscle mass index (SMI), handgrip strength, and gait speed in patients with chronic liver disease. The serum 25(OH)D levels were significantly correlated with the (**A**) SMI (*r* = 0.220, *p* = 0.001), (**B**) handgrip strength (*r* = 0.304, *p* < 0.001), and (**C**) gait speed (*r* = 0.251, *p* < 0.001).

The serum 25(OH)D levels correlated significantly with the following baseline factors: BCAA, handgrip strength, SMI, and gait speed. The correlation coefficients for handgrip strength, SMI, and gait speed were 0.304 (*p* < 0.001), 0.220 (*p* = 0.001), and 0.251 (*p* < 0.001), respectively (Figure 3, Table S4).

## 4. Discussion

Some studies on elderly adults reported that vitamin D deficiency was associated with sarcopenia and impaired physical performance [25,26,27,28], while others reported that low vitamin D levels were related to frailty and mortality [29,30,31,32]. These results suggest that lower serum vitamin D levels are closely related to frailty and sarcopenia in elderly individuals. However, these relationships have not yet been reported and remain unclear in CLD. In this study, we focused on the relationships between serum vitamin D levels and frailty or sarcopenia in patients with CLD and showed that lower serum 25(OH)D levels were significantly and independently related to frailty and sarcopenia in such patients.

However, we failed to confirm a statistically significant relationship between the conventional classification of vitamin D status and frailty or sarcopenia, as most patients were vitamin D deficient. Therefore, we provisionally reclassified the patients into three groups based on the serum 25(OH)D levels and investigated the association between the vitamin D levels and frailty or sarcopenia. In this reclassification, the cutoff value for the L-VD group [25(OH)D ≤10.5 ng/mL] was close to that for patients with “severe” vitamin D deficiency (≤10 ng/mL or ≤12 ng/mL) [16,36]. Notably, the L-VD group had the highest prevalence rate of frailty and sarcopenia, while the H-VD group had the lowest prevalence among the three groups. The prevalence of frailty and sarcopenia increased significantly in a stepwise manner with a decline in the vitamin D status. In addition, the 25(OH)D levels correlated positively and significantly with handgrip strength, SMI, and gait speed. These results suggest that lower serum vitamin D levels, especially severe vitamin D deficient status, are closely related to frailty and sarcopenia in patients with CLD. In addition, the provisional reclassification used in the present study may be useful in clinically treating patients with CLD and frailty and/or sarcopenia.

The vitamin D molecule and vitamin D receptor (VDR) play crucial roles in regulating the musculoskeletal system [24,27,37]. The VDR is expressed in murine and human skeletal muscles, which suggests that vitamin D has direct effects on muscle tissue [27,38,39]. However, the expression level of VDR declines with increasing age [27]. Mice with vitamin D deficiency and global VDR knockout mice showed reduced muscle mass and strength, and muscle decline progressed with age and an increase in the duration of vitamin D deficiency [40]. In addition, both the mouse models showed significant increases in the expression level of myostatin, a member of the transforming growth factor-β family and a negative regulator of muscle protein synthesis [40]. An in vitro study has shown that vitamin D stimulates myogenic cell differentiation by modulating the expression of pro- and anti-myogenic factors [41].

Skeletal muscle fibers are classified into type I (slow-twitch) and type II (fast-twitch) fibers [27,42]. Type I muscle fibers are characterized by low-power production and high endurance capacity, while type II fibers are characterized by high speed and high strength contractions and are important for sprinting exercises [27]. Sarcopenia is characterized by a reduction in the size and proportion of type II fibers [42]. Histological assessment of muscle biopsy specimens from patients with vitamin D deficiency demonstrated a predominance of type II fiber atrophy accompanied by fat cell and glycogen granule infiltration and increased fibrosis [43]. These clinical and basic research findings indicate that both vitamin D deficiency and decreased VDR levels in muscle tissues are associated with sarcopenia and impaired physical performance. Indeed, in the present study, vitamin D deficiency was present in 87.0% (201/231) of the patients. Notably, approximately half of the patients in the L-VD group had both frailty and sarcopenia. These findings suggest that we must pay more careful attention to patients with severe vitamin D deficiency, especially those with CLD. However, this study did not investigate the expression of VDR in muscle tissues and the association between VDR expression levels and frailty/sarcopenia. Future studies should assess whether the levels of circulating vitamin D and VDR expression in muscle tissues are involved with these complications in patients with CLD.

As described previously, a long period of vitamin D deficiency exacerbates muscle weakness. Therefore, maintaining sufficient vitamin D levels is important to prevent the loss of muscle function [40]. A randomized controlled study of stroke survivors demonstrated that supplementation of vitamin D increased the number and size of type II muscle fibers and improved muscle strength, and consequently, reduced the frequency of falls and hip fractures [44]. Furthermore, vitamin D supplementation for four months increased the expression level of intramyonuclear VDR and the size of muscle fibers in older women with vitamin D insufficiency [45]. A systematic review with meta-analysis reported that daily supplementation of vitamin D improved muscle strength and balance in elderly adults, although its effect on gait was not observed [46]. Another meta-analysis of vitamin D supplementation trials showed positive effects on muscle strength; however, the effect was confined to the lower limbs, and vitamin D supplementation had no impact on muscle mass [47]. Hence, the effects of vitamin D supplementation on skeletal muscle mass and strength are controversial. Considering that sarcopenia has a multifactorial etiology, including nutrition, hormonal status, physical activity, and lifestyle, a comprehensive management strategy, including vitamin D supplementation, physical exercise, and awareness of complications, is required to treat and prevent sarcopenia.

This study has some limitations. First, we did not investigate the nutritional intakes and daily activities, including their exposure to sunlight, which might influence the serum 25(OH)D levels. Second, we did not investigate the mental state (including a depressed mood) and the use of antidepressants, which are associated with impaired physical activity and sarcopenia [48,49]. The severity of depression inversely correlates with serum vitamin D levels in patients with CLD [50].

## 5. Conclusions

In this study, we showed that lower serum 25(OH)D levels were significantly and independently related to frailty and sarcopenia in patients with CLD. The prevalence rates of frailty and sarcopenia significantly increased in a stepwise manner with a decline in the vitamin D status (the status was reclassified provisionally). The optimal classification of vitamin D status for patients with CLD may need to be reconsidered to treat those with frailty and/or sarcopenia. Comprehensive assessment (including serum 25(OH)D levels) and early therapeutic interventions (including vitamin D supplementation) are imperative, especially for patients with CLD and severe vitamin D deficiency.

## Figures and Tables

**Table 1:** Baseline clinical characteristics among patients with and without sarcopenia.

Variable	All Patients	Sarcopenia Group	Non-Sarcopenia Group	*p* Value
Patients, n (%)	231	66 (28.6)	165 (71.4)	
Man, n (%)	95 (41.1)	23 (34.8)	72 (43.6)	0.220
Age (years)	70.0 (60.0–76.0)	76.0 (72.8–80.3)	68.0 (58.0–73.0)	<0.001
BMI (kg/m^2^)	23.1 (20.7–26.0)	20.5 (19.2–22.6)	24.2 (21.8–26.6)	<0.001
Liver cirrhosis, n (%)	98 (42.4)	40 (60.6)	58 (35.2)	<0.001
Etiology				
HBV/HCV/PBC/other, n	42/90/60/39)	5/36/19/6	37/54/41/33)	0.002
Total bilirubin (mg/dL)	0.7 (0.5–0.9)	0.6 (0.5–0.9)	0.7 (0.5–0.9)	0.610
Albumin (g/dL)	4.1 (3.9–4.4)	3.9 (3.6–4.3)	4.2 (3.9–4.4)	0.003
Prothrombin time INR	1.02 (0.96–1.11)	1.05 (0.96–1.13)	1.02 (0.96–1.10)	0.130
BCAA (μmol/L)	408 (357–475)	364 (302–425)	432 (381–488)	<0.001
25(OH)D (ng/mL)	14.0 (10.5–18.1)	12.2 (8.8–15.7)	14.7 (11.4–18.4)	0.001
Vitamin D insufficiency, n (%)	26 (11.3)	4 (6.1)	22 (13.3)	0.114
Vitamin D deficiency, n (%)	201 (87.0)	62 (93.9)	139 (84.2)	0.048
SMI (kg/m^2^)				
All patients	6.30 (5.54–7.12)	5.20 (4.82–5.71)	6.75 (5.98–7.44)	<0.001
Man	7.13 (6.48–7.87)	6.18 (5.49–6.84)	7.49 (7.05–8.15)	<0.001
Woman	5.86 (5.20–6.44)	5.09 (4.67–5.30)	6.05 (5.83–6.59)	<0.001
Handgrip strength (kg)				
All patients	22.1 (17.4–29.4)	16.8 (13.8–18.4)	24.5 (19.9–32.6)	<0.001
Man	30.5 (24.1–37.5)	22.8 (18.3–24.2)	33.4 (28.5–38.9)	<0.001
Woman	18.8 (15.1–22.5)	15.0 (12.8–17.2)	21.3 (18.5–24.0)	<0.001
Gait speed (m/s)	1.10 (0.91–1.25)	0.90 (0.68–1.07)	1.15 (1.02–1.28)	<0.001
Slow gait speed, n (%)	81 (35.1)	49 (74.2)	32 (19.4)	<0.001
Frailty, n (%)	70 (30.3)	51 (77.3)	19 (11.5)	<0.001

Values are presented as median (interquartile range) or number (percentage). Statistical analysis was carried out using the chi-squared test or the Mann–Whitney U test, as appropriate. 25(OH)D, 25-hydroxyvitamin D; BCAA, branched-chain amino acid; BMI, body mass index; HBV, hepatitis B virus; HCV, hepatitis C virus; INR, international normalized ratio; PBC, primary biliary cholangitis; SMI, skeletal muscle mass index.

**Table 2:** Significant factors related to sarcopenia in patients with chronic liver disease.

Variable	Univariate		Multivariate	
	OR (95% CI)	*p*-Value	OR (95% CI)	*p* Value
Age (years)	1.083 (1.047–1.121)	<0.001	1.087 (1.044–1.130)	<0.001
BMI (kg/m^2^)	0.726 (0.649–0.813)	<0.001	0.720 (0.635–0.817)	<0.001
Liver cirrhosis	2.838 (1.576–5.111)	0.001	2.493 (1.180–5.266)	0.017
Albumin (g/dL)	0.334 (0.184–0.605)	<0.001		
Prothrombin time INR	11.004 (1.104–109.651)	0.041		
BCAA (μmol/L)	0.992 (0.988–0.995)	<0.001		
25(OH)D (ng/mL)	0.896 (0.841–0.953)	0.001	0.863 (0.794–0.937)	<0.001
Vitamin D deficiency	2.899 (0.971–8.661)	0.057		

25(OH)D, 25-hydroxyvitamin D; BCAA, branched-chain amino acid; BMI, body mass index; CI, confidence interval; INR, international normalized ratio; OR, odds ratio.

**Table 3:** Significant factors related to frailty in patients with chronic liver disease.

Variable	Univariate		Multivariate	
	OR (95% CI)	*p*-Value	OR (95% CI)	*p* Value
Age (years)	1.091 (1.054–1.129)	<0.001	1.094 (1.052–1.138)	<0.001
BMI (kg/m^2^)	0.869 (0.801–0.943)	0.001		
Liver cirrhosis	4.844 (2.645–8.870)	<0.001	3.749 (1.843–7.626)	<0.001
Albumin (g/dL)	0.292 (0.159–0.534)	<0.001		
Prothrombin time INR	18.162 (1.821–181.168)	0.013		
BCAA (μmol/L)	0.991 (0.988–0.995)	<0.001	0.994 (0.990–0.998)	0.008
25(OH)D (ng/mL)	0.893 (0.840–0.950)	0.001	0.887 (0.822–0.957)	0.002
Vitamin D deficiency	2.390 (0.875–6.526)	0.089		

25(OH)D, 25-hydroxyvitamin D; BCAA, branched-chain amino acid; BMI, body mass index; CI, confidence interval; INR, international normalized ratio; OR, odds ratio.

**Table 4:** Characteristics of the three groups classified based on the serum 25-hydroxyvitamin D levels.

Variable	L-VD	M-VD	H-VD	*p* Value
Patients, n (%)	57 (24.7)	115 (49.8)	59 (25.5)	
Man, n (%)	20 (35.1)	41 (35.7)	34 (57.6)	0.012
Age (years)	68.0 (55.0–77.0)	70.0 (61.0–76.0)	72.0 (64.0–77.0)	0.317
BMI (kg/m^2^)	22.2 (20.2–26.0)	22.8 (20.4–26.1)	23.6 (22.3–25.8)	0.489
Liver cirrhosis, n (%)	30 (52.6)	48 (41.7)	20 (33.9)	0.122
Etiology				
HBV/HCV/PBC/other, n	8/21/15/13	20/50/27/18	14/19/18/8	0.499
Total bilirubin (mg/dL)	0.7 (0.5–1.3)	0.6 (0.5–0.8)	0.7 (0.5–0.9)	0.238
Albumin (g/dL)	4.1 (3.7–4.5)	4.1 (3.8–4.3)	4.2 (4.0–4.4)	0.209
Prothrombin time INR	1.06 (0.96–1.16)	1.01 (0.95–1.11)	1.00 (0.97–1.07)	0.139
BCAA (µmol/L)	382 (312–432)	420 (364–478)	438 (379–501)	<0.001
25(OH)D (ng/mL)	8.4 (6.9–9.7)	14.0 (12.4–15.9)	20.1 (18.7–23.5)	<0.001
SMI (kg/m^2^)				
ALL patients	5.87 (5.08–6.96)	6.16 (5.66–6.95)	7.04 (5.97–7.43)	0.002
Man	7.07 (6.44–8.27)	6.98 (6.17–7.76)	7.23 (7.01–8.11)	0.152
Woman	5.39 (4.87–5.94)	5.97 (5.44–6.49)	5.90 (5.16–6.70)	0.014
Handgrip strength (kg)				
ALL patients	18.6 (14.8–25.1)	22.1 (17.8–27.1)	26.9 (20.6–33.2)	<0.001
Man	26.3 (18.3–33.5)	30.2 (23.9–37.0)	32.5 (27.0–38.9)	0.036
Woman	17.4 (14.1–19.9)	19.1 (16.5–22.5)	19.4 (16.7–24.0)	0.082
Sarcopenia, n (%)	28 (49.1)	27 (23.5)	11 (18.6)	<0.001
Gait speed (m/s)	1.00 (0.69–1.16)	1.11 (0.92–1.25)	1.17 (1.00–1.38)	0.003
Slow gait speed, n (%)	29 (50.9)	38 (33.0)	14 (23.7)	0.007
Frailty, n (%)	28 (49.1)	33 (28.7)	9 (15.3)	<0.001

Values are presented as median (interquartile range) or number (percentage). Statistical analysis was carried out using the chi-squared test or the Kruskal–Wallis test, as appropriate. 25(OH)D, 25-hydroxyvitamin D; BCAA, branched-chain amino acid; BMI, body mass index; HBV, hepatitis B virus; HCV, hepatitis C virus; INR, international normalized ratio; PBC, primary biliary cholangitis; SMI, skeletal muscle mass index.

## Supplementary Materials

The following are available online at https://www.mdpi.com/2072-6643/12/12/3810/s1, Figure S1: Classification based on the baseline serum 25-hydroxyvitamin D levels, Table S1: Univariate analysis for significant factors related to sarcopenia, Table S2: Comparison of clinical characteristics among patients with and without frailty, Table S3: Univariate analysis for significant factors related to frailty, Table S4: Correlations between 25-hydroxyvitamin D concentrations and baseline characteristics.
