# Supplementary material for: Low Serum 25-Hydroxyvitamin D Levels Are Related to Frailty and Sarcopenia in Patients with Chronic Liver Disease
Source: Nutrients. 2020 Dec 12;12(12):3810. doi: 10.3390/nu12123810 (PMC7764249; doi:10.3390/nu12123810)
Supplement: Supplementary file 1 [file nutrients-12-03810-s001.pdf]

Supplementary Materials

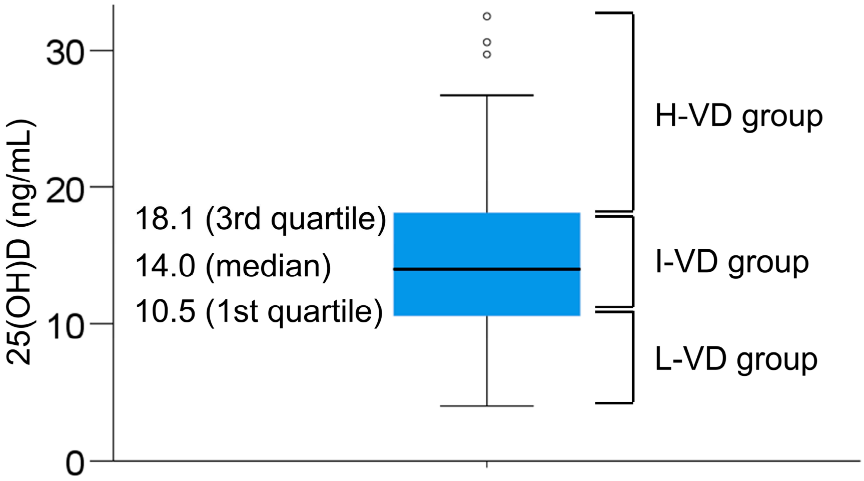

Figure S1. Classification based on the baseline serum 25-hydroxyvitamin D [25(OH)D] levels. The median (interquartile range) 25(OH)D levels was 14.0 (10.5–18.1) ng/mL. The 231 patients were classified into three groups: (1) low 25(OH)D (L-VD) group with ≤10.5 ng/mL (first quartile); (2) intermediate 25(OH)D (I-VD) group with between 10.5 and 18.1 ng/mL (third quartile); and (3) high 25(OH)D (H-VD) group with ≥18.1 ng/mL.

Table S1. Univariate analysis for significant factors related to sarcopenia.

Variable	OR (95% CI)	*p* value
Gender (Man)	0.691(0.382–1.249)	0.221
Age (years)	1.083(1.047–1.121)	< 0.001
BMI (kg/m^2)	0.726(0.649–0.813)	< 0.001
Liver cirrhosis	2.838(1.576–5.111)	0.001
Etiology	0.952(0.778–1.164)	0.630
Total bilirubin (mg/dL)	1.372(0.894–2.106)	0.148
Albumin (g/dL)	0.334(0.184–0.605)	< 0.001
Prothrombin time INR	11.004(1.104–109.651)	0.041
BCAA (μmol/L)	0.992(0.988–0.995)	< 0.001
25(OH)D (ng/mL)	0.896(0.841–0.953)	< 0.001
Vitamin D insufficiency	0.419(0.139–1.268)	0.124
Vitamin D deficiency	2.899(0.971–8.661)	0.057

25(OH)D, 25-hydroxyvitamin D; BCAA, branched-chain amino acid; BMI, body mass index; CI, confidence interval; INR, international normalized ratio; OR, odds ratio.

Table S2. Comparison of clinical characteristics among patients with and without frailty.

Variable	Frailty group	Non-frailty group	*p* value
Patients, n (%)	70 (30.3)	161 (69.7)	
Man, n (%)	25 (35.7)	70 (43.5)	0.270
Age (years)	76.0 (72.8–81.0)	68.0 (58.0–73.0)	< 0.001
BMI (kg/m^2)	21.4 (19.8–24.5)	23.8 (21.4–26.4)	< 0.001
Liver cirrhosis, n (%)	48 (68.6)	50 (31.1)	< 0.001

Etiology			
HBV/HCV/PBC/other, n	7/37/15/11	35/53/45/28	0.023
Total bilirubin (mg/dL)	0.6 (0.5–0.9)	0.7 (0.5–0.9)	0.494
Albumin (g/dL)	3.9 (3.5–4.3)	4.2 (3.9–4.4)	< 0.001
Prothrombin time INR	1.06 (0.99–1.14)	1.01 (0.95–1.09)	0.014
BCAA (μmol/L)	380 (310–419)	434 (375–490)	< 0.001
25(OH)D (ng/mL)	12.2 (9.0–15.0)	14.9 (11.4–18.6)	< 0.001
Vitamin D insufficiency	5 (7.1)	21 (13.0)	0.192
Vitamin D deficiency, n (%)	65 (92.9)	136 (84.5)	0.081
SMI (kg/m^2)			
All patients	5.44 (4.88–6.18)	6.60 (5.97–7.44)	< 0.001
Man	6.18 (5.57–6.84)	7.49 (6.99–8.16)	< 0.001
Woman	5.17 (4.71–5.74)	5.99 (5.58–6.57)	< 0.001
Handgrip strength (kg)			
All patients	16.0 (12.7–19.3)	24.5 (20.3–32.8)	< 0.001
Man	22.8 (18.3–24.6)	33.9 (29.4–39.1)	< 0.001
Woman	14.6 (11.1–16.9)	21.3 (18.3–24.0)	< 0.001
Gait speed (m/s)	0.78 (0.64–0.93)	1.17 (1.08–1.30)	< 0.001
Slow gait speed, n (%)	65 (92.9)	16 (9.9)	< 0.001
Sarcopenia, n (%)	51 (72.9)	15 (9.3)	< 0.001

Values are presented as median (interquartile range) or number (percentage). Statistical analysis was carried out using the chi-squared test or the Mann-Whitney U test, as appropriate. 25(OH)D, 25-hydroxyvitamin D; BCAA, branched-chain amino acid; BMI, body mass index; HBV, hepatitis B virus; HCV, hepatitis C virus; INR, international normalized ratio; PBC, primary biliary cholangitis; SMI, skeletal muscle mass index.

Table S3. Univariate analysis for significant factors related to frailty.

Variable	OR (95% CI)	*p* value
Gender (Man)	0.722(0.404–1.290)	0.271
Age (years)	1.091(1.054–1.129)	< 0.001
BMI (kg/m^2)	0.869(0.801–0.943)	0.001
Liver cirrhosis	4.844(2.645–8.870)	< 0.001
Etiology	0.969(0.795–1.181)	0.756
Total bilirubin (mg/dL)	1.256(0.829–1.904)	0.282
Albumin (g/dL)	0.292(0.159–0.534)	< 0.001
Prothrombin time INR	18.162(1.821–181.168)	0.013
BCAA (μmol/L)	0.991(0.988–0.995)	< 0.001
25(OH)D (ng/mL)	0.893(0.840–0.950)	< 0.001
Vitamin D insufficiency	0.513(0.185–1.420)	0.199
Vitamin D deficiency	2.390(0.875–6.526)	0.089

25(OH)D, 25-hydroxyvitamin D; BCAA, branched-chain amino acid; BMI, body mass index; CI, confidence interval; INR, international normalized ratio; OR, odds ratio.

Table S4. Correlations between 25-hydroxyvitamin D concentrations and baseline characteristics.

Variable	Correlation coefficient	*p* value
Age (years)	0.099	0.133
BMI (kg/m^2)	0.053	0.419
Total bilirubin (mg/dL)	-0.033	0.617
Albumin (g/dL)	0.113	0.086
Prothrombin time INR	-0.111	0.092
BCAA (μmol/L)	0.268	< 0.001
SMI (kg/m^2)	0.220	0.001

Handgrip strength (kg)	0.304	< 0.001
Gait speed (m/s)	0.251	< 0.001

BCAA, branched-chain amino acid; BMI, body mass index; INR, international normalized ratio; SMI, skeletal muscle mass index.
